# Supplemental CO_2_ improves oxygen saturation, oxygen tension_,_ and cerebral oxygenation in acutely hypoxic healthy subjects

**DOI:** 10.14814/phy2.14513

**Published:** 2020-07-29

**Authors:** Jan Stepanek, Ryan A. Dunn, Gaurav N. Pradhan, Michael J. Cevette

**Affiliations:** ^1^ Mayo Clinic Aerospace Medicine and Vestibular Research Laboratory (AMVRL) Scottsdale AZ USA; ^2^ Mayo Clinic Alix School of Medicine Scottsdale AZ USA

**Keywords:** cerebral perfusion, CO_2_ supplementation, hypoxia, oxygenation

## Abstract

Oxygen is viewed in medicine as the sole determinant of tissue oxygenation, though carbon dioxide homeostasis is equally important and clinically often ignored. The aims of this study were as follows: (a) to examine the effects of different acute hypoxic conditions on partial pressure of arterial oxygen (${\text{Pa}}_{O_{2}}$), arterial oxygen saturation of hemoglobin (${\text{Sa}}_{O_{2}}$), and regional cerebral saturation of hemoglobin (rSO_2_); and (b) to evaluate supplemental CO_2_ as a tool to improve oxygenation in acutely hypoxic individuals. We hypothesized that exposure to gas mixtures with added CO_2_ would improve oxygenation in hypoxic human subjects. Twenty healthy subjects were exposed to 5‐min intervals of two gas mixtures: hypoxic gas mixture containing 8% oxygen, and a CO_2_‐enriched mixture containing 8% oxygen plus either 3% or 5% CO_2_. Ten subjects received the 3% CO_2_‐enriched mixture, and the remaining 10 subjects received the 5% CO_2_‐enriched mixture. The order of exposure was randomized. Blood gases, pulse oximetry, end‐tidal CO_2_, and cerebral oximetry were measured. Compared to the purely hypoxic gas group, ${\text{Pa}}_{O_{2}}$ was increased in the 3% and 5% CO_2_‐enriched groups by 14.9 and 9.5 mmHg, respectively. Compared to pure hypoxia, ${\text{Sa}}_{O_{2}}$ was increased in the 3% and 5% CO_2_‐enriched groups by 16.8% and 12.9%, respectively. Both CO_2_‐enriched gas groups had significantly higher end‐exposure rSO_2_ and recovered to baseline rSO_2_ within 1 min, compared to the pure hypoxic gas group, which returned to baseline in 5 min. These results suggest that in acutely hypoxic subjects, CO_2_ supplementation improves blood oxygen saturation and oxygen tension as well as cerebral oxygenation measures.

## Graphical abstract

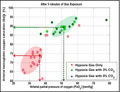

## 1 INTRODUCTION

Supplemental oxygen systems have been utilized in aviation and mountaineering for many years, though such solutions address only oxygen supply and not carbon dioxide (capnic) homeostasis. At altitude, decreased atmospheric partial pressure of oxygen (P_O2_) triggers increases in ventilation, increasing the rates of both oxygen uptake and CO_2_ elimination (West, 2012). The resulting hypocapnia is generally well‐tolerated in healthy individuals. Hypocapnia is a critical and often overlooked detriment to optimal tissue oxygenation. Hypocapnia may decrease oxygenation by several mechanisms including increases in pulmonary ventilation‐perfusion mismatch, vasoconstriction in many vascular beds, and diminished oxygen unloading from hemoglobin due to the left shift in the oxy‐hemoglobin dissociation curve (Domino, Lu, Eisenstein, & Hlastala, 1993; Gustafsson, Sjoberg, Lewis, & Thorborg, 1993; Naeraa, Petersen, Boye, & Severinghaus, 1966). Hypocapnia concomitantly increases cellular oxygen demand via increased glycolysis, mitochondrial respiration, and sympathoadrenal tone, thereby exacerbating oxygen supply demand mismatch in hypoxic individuals (Laffey & Kavanagh, 2002). Therefore, it is prudent to consider both CO_2_ and O_2_ homeostasis in individuals who operate in hypoxic austere environments.

Few prior studies have examined hypocapnia as a contributor to hypoxia. Mosso's, 1898 experiments were the first to administer supplemental CO_2_ to hypoxic subjects in hypobaric chambers and in field studies in the Alps (Mosso, 1898). Over the next several decades, four more studies demonstrated a relationship between normocapnia and optimal oxygenation at altitude with a primary interest in improving oxygenation at high altitude (Childs, Hamlin, & Henderson, 1935; Douglas & Haldane, 1909; Douglas, Haldane, Henderson, & Schneider, 1913; Henderson, 1938). In 1988, TC Harvey demonstrated in an uncontrolled study that administration of supplemental CO_2_ improved acute mountain sickness symptoms in mountaineers (Harvey et al., 1988). Studies by Bärtsch and Maher cast doubt on these findings, as neither found beneficial effects of supplemental CO_2_ (Bartsch, Baumgartner, Waber, Maggiorini, & Oelz, 1990; Maher et al., 1975). Two recent studies have examined supplemental inspired CO_2_ as a tool to increase oxygenation and performance. In 2003, Imray et al. studied subjects at 150 m and at 3459 m breathing 3% CO_2_, 35% oxygen plus 3% CO_2_, and 35% oxygen, and measured effects on cerebral and peripheral oxygenation after breathing the gases for 5 min. At sea level and at altitude, they observed markedly increased arterial blood oxygen and cerebral oxygenation in subjects breathing the mixture of CO_2_ and oxygen (Imray et al., 2003). In 2007, Van Dorp et al. demonstrated that subjects with mild to moderate hypoxia performed better on vigilance and task performance tests following inhalation of gas mixtures with added CO_2_ (Van Dorp et al., 2007). Ashkanian et al. found that inhalation of 5% CO_2_ increased cerebral blood flow and cerebral oxygenation in normoxic individuals (Ashkanian, Borghammer, Gjedde, Ostergaard, & Vafaee, 2008). Physiologically the maintenance of normal oxygen and normal CO_2_ levels are critical to optimal tissue oxygen delivery.

We hypothesized that breathing a gas mixture of 8% oxygen supplemented with CO_2_ (3% and 5%) would improve oxygenation in hypoxic subjects when compared with breathing a gas mixture of 8% oxygen. The aims of this study were to examine the effects of inhaled hypoxic gas enriched with CO_2_ on partial pressure of arterial oxygen (${\text{Pa}}_{O_{2}}$), oxygen saturation of hemoglobin (${\text{Sa}}_{O_{2}}$), and regional cerebral saturation of hemoglobin (rSO_2_).

## 2 METHODS

The study protocol was approved by the Mayo Clinic Institutional Review Board. Volunteer subjects were selected from a healthy volunteer subject pool at Mayo Clinic for inclusion in the study. To avoid hypoxic collapse (loss of consciousness), the exposure time was limited to 5 min. Exclusion criteria included age <18 years and any history of obstructive or restrictive pulmonary disease, cardiovascular disease, epilepsy, diabetes mellitus, chronic headaches, neurological disorders, and hematological disorders. A negative urine pregnancy test was required for female subjects. Informed consent was obtained from all subjects prior to enrollment.

### 2.1 Equipment and procedures

Protocols were performed at the Aerospace Medicine and Vestibular Research Laboratory at the Mayo Clinic in Arizona (altitude 500 m [1,640 ft]; ambient pressure 716 mmHg). Subjects donned a Gentex military helmet and aviator tight‐fit facemask throughout the experimental protocols. Prior to initiation of the protocol, the mask hose was occluded at the inlet and the subjects were asked to inhale, effectively demonstrating a tight seal to ensure the absence of significant gas leaks. End‐tidal CO_2_ was recorded at the exhalation port of the aviator mask through a capnographic device (LifeSense^®^ Tabletop Capnography, Nonin Medical, Inc.). Cerebral oximetry (rSO_2_) was recorded via Nonin Equanox 7600. The Equanox dual emitter and dual detector sensor (8000CA) was attached to the left forehead to measure rSO_2_.

Three different hypoxic conditions were induced by provision of premixed gas mixtures through the aviator mask. The acute hypoxic condition was simulated by use of a gas mixture containing 8% oxygen, balance nitrogen. The two supplemental CO_2_ conditions were induced by use of gas mixtures containing 8% oxygen, either 3% or 5% CO_2_, and balance nitrogen. Normoxia was achieved by providing ambient air through the mask. This study did not record measures of ventilation.

All baseline data were recorded under normoxic conditions prior to gas administration. For initial gas mixture exposure, subjects were randomized to either pure hypoxia or hypoxia with added CO_2_ for 5 min. This was followed by a 20‐min normoxia washout period, then administration of the alternative protocol. Ten subjects received 3% CO_2_ and 8% oxygen, balance nitrogen mixes. The remaining 10 subjects received 5% CO_2_ and 8% oxygen, balance nitrogen gas. Data were recorded at 1‐min intervals during each 5‐min gas exposure. Additionally, arterial blood gas analysis (ABGA) was performed at the end of each 5‐min gas exposure. The arterial blood was obtained by radial artery puncture by standard technique and immediately analyzed on an automated blood gas analysis machine (Radiometer ABL80 with cooximetry).

### 2.2 Statistical analysis

Data analysis followed collection using descriptive statistics, Student's *t* tests, and repeated‐measures ANOVA. All results are presented as means ± *SD*.

## 3 RESULTS

A sample of 20 subjects (men:women, 10:10) was enrolled. Subject demographics showed values of: age (35 ± 9.2 years, range 22–51), height (172.4 ± 9.1 cm), weight (76.6 ± 15.5 kg), and BMI (25.7 ± 4.7). There was no significant difference in age or BMI range or mean values by sex. Subjects were healthy, denied use of any medications, and were all nonsmokers. All 20 subjects completed the entire experimental protocol, including recordings at baseline, during exposure to the different hypoxic gas mixtures, and during the two post‐exposure normoxia periods.

**FIGURE 1:**
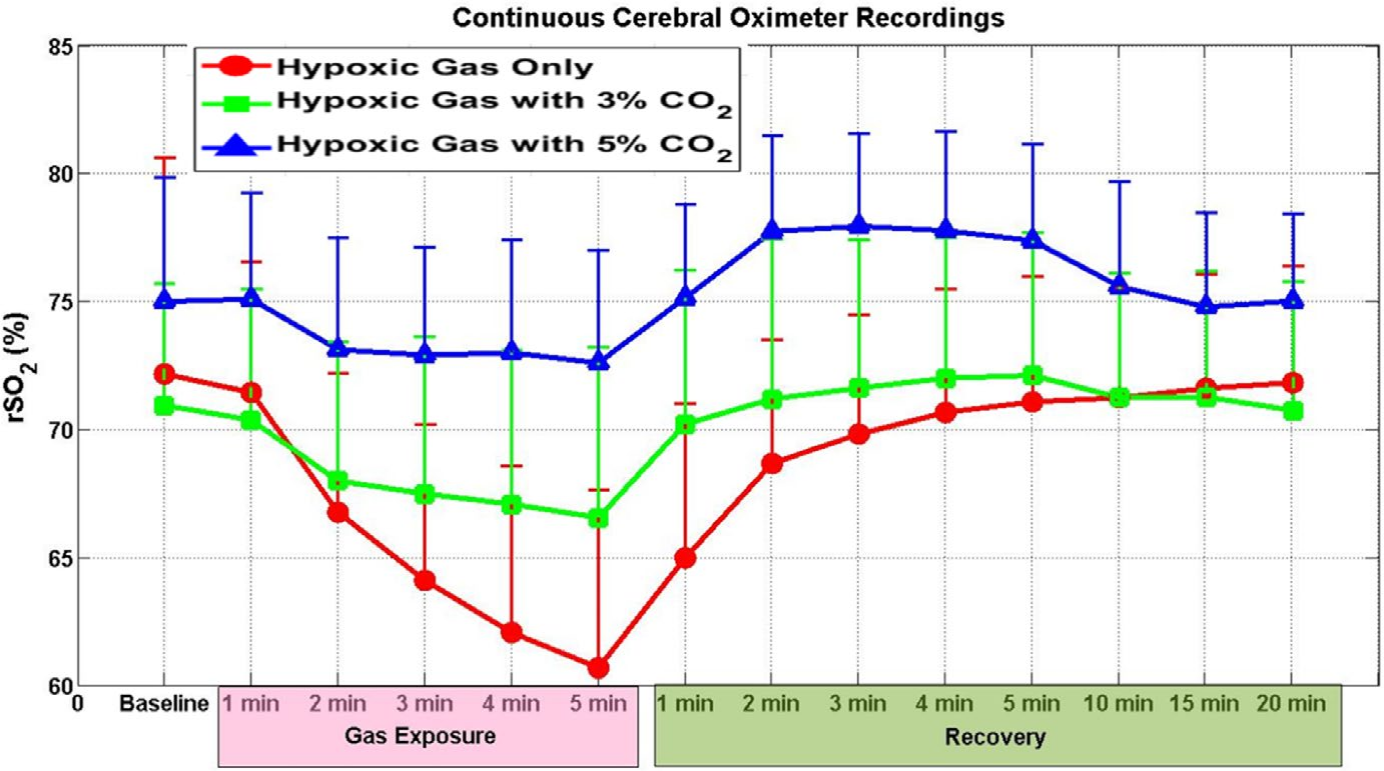
Continuous cerebral oximetry (means ± *SD*) by exposure time. Note that both capnic exposures (3% and 5%) demonstrate significantly improved rSO_2_ compared to hypoxic conditions alone. This improvement is more pronounced and longer lasting in the 5% group, persisting until 10 min into normoxic recovery conditions

Baseline mean rSO_2_ values ranged from 63%–82.3%, with 73.3 ± 4.9%. There was no significant difference between rSO_2_ baseline values before the different exposures. Experimental rSO_2_ values are shown in Figure 1. The purely hypoxic gas exposure yielded the lowest rSO2 values (rSO_2_ = 60.7 ± 6.9%). The 3% added CO_2_ group was found to have rSO_2_ values higher than their relative pure hypoxic gas exposure values after 4 min, persisting with significant elevation after 5 min of exposure (rSO_2_ = 66.5 ± 6.7%) and after 1 min of recovery in normoxic conditions. By the second minute of recovery, there was no significant difference in rSO_2_ between pure hypoxic gas and 3% CO_2_‐enriched gas groups. The 5% CO_2_‐enriched gas group had rSO_2_ values significantly higher than their relative purely hypoxic gas values after 2 min of exposure, persisting with significant elevation after 5 min of exposure (rSO_2_ = 72.6 ± 4.3%) and for the first 5 min of recovery in normoxic conditions. By 10 min of recovery, there was no significant difference between rSO_2_ in the purely hypoxic gas values compared to the 5% CO_2_‐enriched gas values.

**FIGURE 2:**
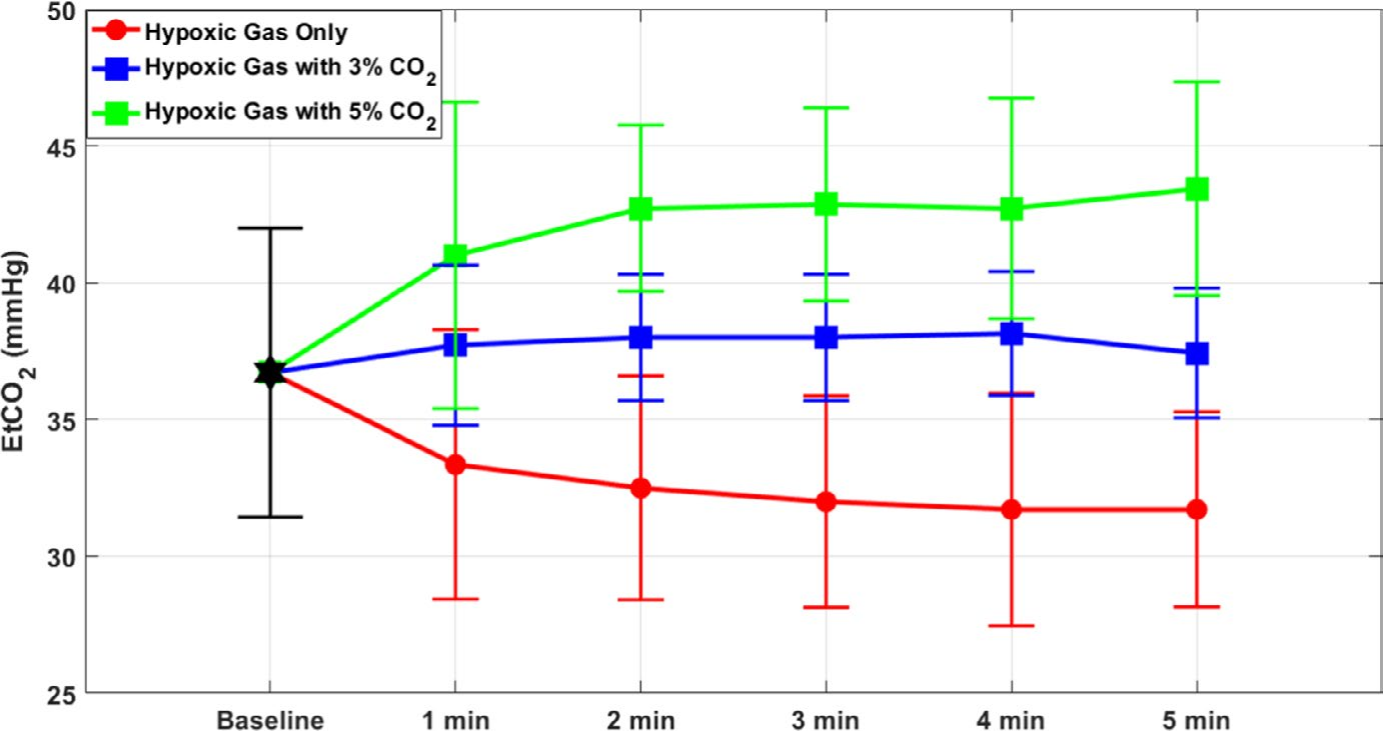
End‐tidal CO_2_ (${\text{ET}}_{{\text{CO}}_{2}}$) measured at 1‐min intervals during gas exposure (means ± *SD*). ${\text{ET}}_{{\text{CO}}_{2}}$ correlates with concentration of CO_2_ in the inhaled gas mixture. Note that each group reaches ${\text{ET}}_{{\text{CO}}_{2}}$ steady state at 1 min

End‐tidal CO_2_ measured at 1‐min intervals over 5 min of exposure demonstrated consistent values across the three groups, with steady state reached by 2 min into the exposure. The 5% CO_2_‐enriched gas group had the highest ${\text{ET}}_{{\text{CO}}_{2}}$, followed by the 3% CO_2_‐enriched gas group, and the pure hypoxia group with the lowest. As expected, each group's ${\text{ET}}_{{\text{CO}}_{2}}$ correlated with the percentage of supplemental inspired CO_2_. A comparison of ${\text{ET}}_{{\text{CO}}_{2}}$ is demonstrated in Figure 2 below.

**FIGURE 3:**
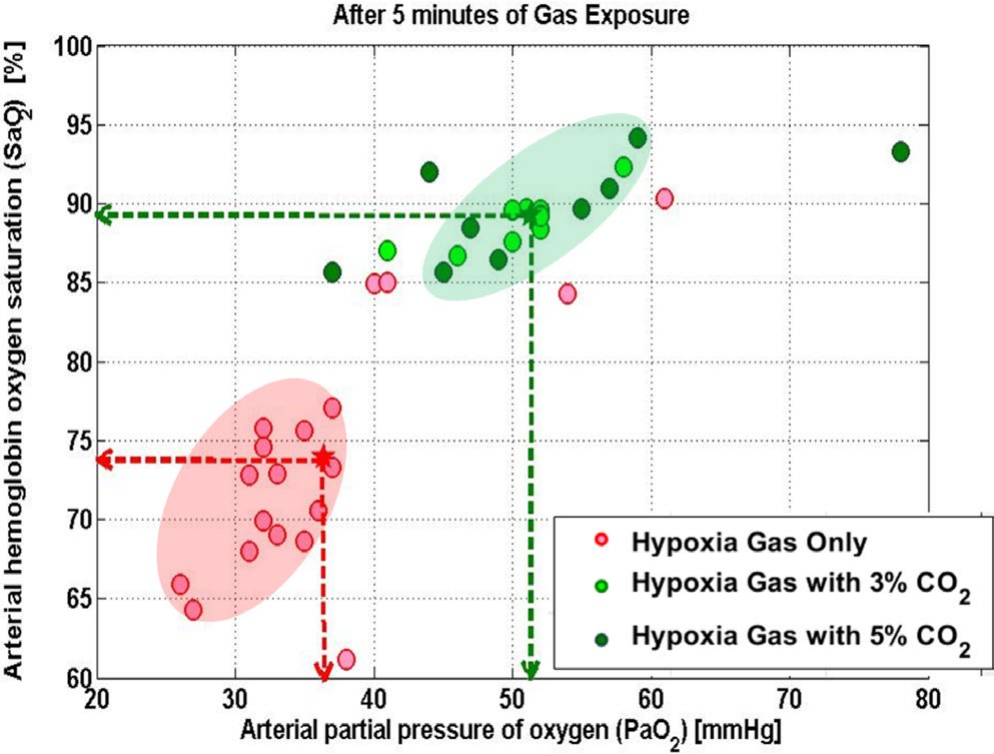
Arterial hemoglobin oxygen saturation (${\text{Sa}}_{O_{2}}$) and arterial partial pressure of oxygen (${\text{Pa}}_{O_{2}}$) after 5 min of gas exposure measured by arterial blood gas analysis. Note that both 3% and 5% CO_2_‐enriched gas groups demonstrate significantly higher ${\text{Sa}}_{O_{2}}$ and ${\text{Pa}}_{O_{2}}$ compared to purely hypoxic conditions

Figure 3 illustrates ${\text{Sa}}_{O_{2}}$ and ${\text{Pa}}_{O_{2}}$ measured at the end of a 5‐min gas mixture exposure. ${\text{Pa}}_{O_{2}}$ in the 3% CO_2_‐enriched gas group increased to 50.4 mmHg from 35.5 mmHg hypoxic gas baseline. ${\text{Pa}}_{O_{2}}$ in the 5% CO_2_‐enriched gas increased to 46.8 mmHg from 37.3 mmHg hypoxic gas baseline. ${\text{Pa}}_{O_{2}}$ values were elevated in the CO_2_‐enriched gas compared to pure hypoxic gas values (pure hypoxic gas mix ${\text{Pa}}_{O_{2}}$ = 35.5 ± 2.7%, 3% CO_2_‐enriched gas ${\text{Pa}}_{O_{2}}$ = 50.4 ± 4.4%, *df* = 18, *p* < .001; pure hypoxic ${\text{Pa}}_{O_{2}}$ = 37.3 ± 12.3%, 5% CO_2_‐enriched gas ${\text{Pa}}_{O_{2}}$ = 46.8 ± 7.2%, *df* = 13, *p* = .05).

${\text{Sa}}_{O_{2}}$ improvements paralleled changes in ${\text{Pa}}_{O_{2}}$. ${\text{Sa}}_{O_{2}}$ in the 3% CO_2_‐enriched gas group increased to 88.9% from 72.1% in the hypoxic gas. ${\text{Sa}}_{O_{2}}$ in the 5% CO_2_‐enriched gas increased to 88.8% from 75.9% hypoxic gas. Overall, ${\text{Sa}}_{O_{2}}$ was elevated in both the 3% and 5% CO_2_‐enriched gas groups compared to hypoxic gas (pure hypoxia ${\text{Sa}}_{O_{2}}$ = 72.12 ± 6.3%, 3% CO_2_‐enriched gas ${\text{Sa}}_{O_{2}}$ = 88.9 ± 1.6, *df* = 18, *p* < 0.001; pure hypoxic gas mix ${\text{Sa}}_{O_{2}}$ = 75.9 ± 9.0%, 5% CO_2_‐enriched gas ${\text{Sa}}_{O_{2}}$ = 88.8 ± 3.8%, *df* = 13, *p* = 0.002).

## 4 DISCUSSION

The administration of 3% or 5% CO_2_‐enriched hypoxic gas mixtures (8% inhaled oxygen) improved ${\text{Sa}}_{O_{2}}$, ${\text{Pa}}_{O_{2}}$, and rSO_2_ of hypoxic individuals in the laboratory setting compared to purely hypoxic stress (Figure 3).

Cerebral oximetry profiles varied markedly between the three exposures (Figure 1). Both CO_2_‐enriched gas groups demonstrated rSO_2_ profiles that were more favorable than purely hypoxic gas, with the 5% CO_2_‐enriched gas group demonstrating the highest overall end‐exposure rSO_2_. Both CO_2_‐enriched gas groups returned to baseline within 2 min of normoxic recovery, whereas the hypoxia only group required 5 min to return to baseline. Interestingly, both CO_2_ supplemented groups exceeded baseline rSO_2_ during the recovery phase, whereas the purely hypoxic gas group did not. This suggests that, compared to hypoxic gas alone, gas with added CO_2_ improves cerebral oxygenation during the hypoxic exposure (Imray et al., 2003) and leads to a much more rapid recovery when normoxia is restored. However, it is important to note that it is unclear if this increase in cerebral oxygenation results in metabolic or functional changes. Pre‐exposure rSO_2_ baselines of the three groups also showed variability, though all matched their post‐exposure baselines. We believe that this variability was a result of smaller samples in the CO_2_‐enriched gas groups, and we emphasize that the hypoxia only group mean approximately equals the mean of the combined 3% and 5% CO_2_‐enriched gas groups, as would be statistically expected.

The response dynamics of cerebral tissue oximetry may be explained by the more complex biological nature of CO_2_ alterations. While changes in oxygen are mediated in the body exclusively by hemoglobin binding (and to a minimal extent plasma and myoglobin), CO_2_ is bound to hemoglobin, bound to proteins, and converted to bicarbonate. Hence, changes in CO_2_ will depend on more than just the rapid interaction with hemoglobin (as is the case with oxygen), but rather also depend on alterations in bicarbonate, plasma protein binding, and tissue binding, and by consequence require a longer time to compensate (Farhi & Rahn, 1955). The maintenance of capnic homeostasis by supplementing inhaled CO_2_ therefore appears to afford the cerebral tissue a more rapid recovery mechanism.

Our results expand upon the findings of Ashkanian and Imray, which reported improved ${\text{Sa}}_{O_{2}}$ with CO_2_ supplementation in normoxic individuals (Ashkanian et al., 2008; Imray et al., 2003). We found the same effect, though we tested this in acutely normobaric hypoxic individuals rather than normoxic or hypobaric hypoxic individuals. Harvey et al. found higher ${\text{Pa}}_{O_{2}}$ in hypoxic individuals following supplemental CO_2_ inhalation, a finding that was mirrored in our data (Harvey et al., 1988). Our findings of improved rSO_2_ with CO_2_‐enriched gas exposure may explain the findings of improvement in cognitive function of Van Dorp et al. (2007).

Our results support the argument that oxygenation requires maintenance of normal oxygen and CO_2_ levels. The discussion of oxygenation tends to center on oxygen alone, while little attention is given to the equally important component of carbon dioxide homeostasis (Swenson, 2019). We hypothesize that supplemental CO_2_ may improve oxygenation by several mechanisms. Foremost, hypocapnia impairs respiratory efficiency by increasing small airway resistance (Croxton, Lande, & Hirshman, 1995) and reducing lung compliance (Cutillo, Omboni, Perondi, & Tana, 1974), thereby increasing ventilation‐perfusion mismatch, as has been demonstrated in hyperventilated dogs (Domino, Swenson, et al., 1993). Hypocapnia induces systemic vasoconstriction in many vascular beds including the brain (Gustafsson et al., 1993), thereby impairing blood flow to tissues. Hypocapnia also increases intrapulmonary shunting due to hypoxic vasoconstriction (Domino, Lu, et al., 1993) and decreases oxygen offloading at the tissues due to a left shift in the oxygen‐hemoglobin dissociation curve (Naeraa et al., 1966). Supplemental inspired CO_2_ may ameliorate these deleterious effects of hypocapnia, thereby improving V/Q mismatch, increasing effective ventilatory surface area, and improving oxygenation. Our results demonstrate the previously discussed physiologic effects of supplemental inhaled CO_2_, ultimately leading to improved oxygenation with supplemental inhaled CO_2_. Taken as a whole, these results suggest that supplemental inhaled CO_2_ with or without supplemental added O_2_ may be of value as a tool to improve oxygenation in individuals operating in hypoxic environments.

### 4.1 Limitations

The degree of hypoxia was of significant severity such that we had to limit the duration of exposure to avoid inherent hypoxic collapse. As a result, the reported data represent the state of the individual subjects after 5 min and are not representative of a true steady state from a respiratory physiology perspective.

The absence of objective measures of ventilation is a limitation of this work and as such it is difficult to differentiate the direct effect of CO_2_ on gas exchange as opposed to the effect of CO_2_ as a respiratory stimulant. Clinically, the experimenters did not perceive any significant difference in breathing patterns among subjects.

One additional potential limitation of this study design is subtle mask leak, which is a difficult variable to entirely eliminate, though it was addressed to the best of our abilities by an initial test to ascertain proper mask fit. We assessed for excessive ventilatory variation among individuals by measuring ${\text{ET}}_{{\text{CO}}_{2}}$ at 1‐min intervals during gas exposure, which we found to be markedly consistent between subjects within gas mixture groups. Across all subjects, end‐tidal CO_2_ reached steady state within 1 min of exposure and remained constant for the remainder of the exposure, as is demonstrated in Figure 2. We interpret this data to indicate limited intersubject variability in minute ventilation and appropriate mask fit. We did not measure ventilation in this study, hence quantification of differences in ventilation beyond the measured ${\text{ET}}_{{\text{CO}}_{2}}$ cannot be provided. An important effect of adding CO_2_ to respired gas is the increase in ventilation due to the respiratory stimulant effect of CO_2_, which also will increase oxygenation to a certain extent (Imray et al., 2000). Additionally, one Pa_CO2_ level and three ${\text{Pa}}_{O_{2}}$ levels were not readable due to technical errors.

## CONFLICT OF INTEREST

None declared.

## AUTHORS' CONTRIBUTIONS

J.S. and G.P. conceived and designed research, wrote IRB and protocol, performed the experiments, and edited the manuscript. R.D. wrote the manuscript with exception of methods and results. G.P. performed the statistical analysis and wrote the methods and results sections of the manuscript. M.C. conceived and designed research, wrote IRB and protocol, and performed the experiments.
